# A randomized controlled trial of interventions to enhance patient-physician partnership, patient adherence and high blood pressure control among ethnic minorities and poor persons: study protocol NCT00123045

**DOI:** 10.1186/1748-5908-4-7

**Published:** 2009-02-19

**Authors:** Lisa A Cooper, Debra L Roter, Lee R Bone, Susan M Larson, Edgar R Miller, Michael S Barr, Kathryn A Carson, David M Levine

**Affiliations:** 1 Welch Center for Prevention, Epidemiology and Clinical Research, Johns Hopkins University, Baltimore, Maryland, USA; 2 Department of Medicine, Johns Hopkins University School of Medicine, Baltimore, Maryland, USA; 3 Department of Epidemiology, Johns Hopkins Bloomberg School of Public Health, Baltimore, Maryland, USA; 4 Department of Health Policy and Management, Johns Hopkins Bloomberg School of Public Health, Baltimore, Maryland, USA; 5 Department of Health, Behavior and Society, Johns Hopkins Bloomberg School of Public Health, Baltimore, Maryland, USA; 6 American College of Physicians, Practice Advocacy and Improvement Division, Washington, DC, USA

## Abstract

**Background:**

Disparities in health and healthcare are extensively documented across clinical conditions, settings, and dimensions of healthcare quality. In particular, studies show that ethnic minorities and persons with low socioeconomic status receive poorer quality of interpersonal or patient-centered care than whites and persons with higher socioeconomic status. Strong evidence links patient-centered care to improvements in patient adherence and health outcomes; therefore, interventions that enhance this dimension of care are promising strategies to improve adherence and overcome disparities in outcomes for ethnic minorities and poor persons.

**Objective:**

This paper describes the design of the Patient-Physician Partnership (Triple P) Study. The goal of the study is to compare the relative effectiveness of the patient and physician intensive interventions, separately, and in combination with one another, with the effectiveness of minimal interventions. The main hypothesis is that patients in the intensive intervention groups will have better adherence to appointments, medication, and lifestyle recommendations at three and twelve months than patients in minimal intervention groups. The study also examines other process and outcome measures, including patient-physician communication behaviors, patient ratings of care, health service utilization, and blood pressure control.

**Methods:**

A total of 50 primary care physicians and 279 of their ethnic minority or poor patients with hypertension were recruited into a randomized controlled trial with a two by two factorial design. The study used a patient-centered, culturally tailored, education and activation intervention for patients with active follow-up delivered by a community health worker in the clinic. It also included a computerized, self-study communication skills training program for physicians, delivered via an interactive CD-ROM, with tailored feedback to address their individual communication skills needs.

**Conclusion:**

The Triple P study will provide new knowledge about how to improve patient adherence, quality of care, and cardiovascular outcomes, as well as how to reduce disparities in care and outcomes of ethnic minority and poor persons with hypertension.

## Background

A compelling amount of evidence documents ethnic disparities in health care and outcomes in the United States [1]. Additionally, there is an inverse relationship between socioeconomic status and health: the lower the socioeconomic status, the higher the risk of morbidity and mortality from chronic disease [2,3]. It is uncertain how much of these differences in health care and outcomes can be explained by environmental, economic and social factors, access to appropriate and effective health and social services, or behavioral risk factors [4]. Health care professionals, researchers, and policymakers in the United States have believed for some time that access to care is the centerpiece in the elimination of disparities in health for racial, ethnic, and social class groups [5-8]. However, differences in traditional barriers of access (such as socioeconomic status and health insurance coverage) between patients only partially explain the observed differences in health care [5,9,10]. Other patient factors that may play an important role include patients' illness beliefs and behavior [11-13], their degree of self-efficacy regarding taking care of their health [14], language barriers [15,16], low health literacy [17,18], preferences for care [19-21], and their level of involvement in medical decision-making [22,23]. All of these patient factors contribute to patients' adherence to recommended therapies. Physician factors that may play a role in disparities in care include self-efficacy regarding care of ethnically and socially diverse patient populations, communication style (*e.g*., patient-centeredness) [24,25], and biases in medical decision-making (intentional or unintentional) [26,27]. Health system factors other than reimbursement or payer status that may contribute to disparities in care include the degree of organizational focus on quality [28], patient concerns [29-31], and cultural competence [11,32-34].

A recent review of the literature reveals that there are few rigorously designed studies to determine which provider-directed strategies are most effective in reducing disparities in healthcare quality between minority and white populations, and that most of the studies that exist do not target conditions, such as cardiovascular disease, known to be a source of health disparities, nor do they collect adequate data to link evidence-based healthcare processes with patient outcomes [35]. Moreover, few studies have simultaneously intervened to train patients to engage more fully in the health care process while also providing physicians with communication skills training to elicit, activate, and support patient participation in the care dialogue. Because hypertension disproportionately affects ethnic minorities and persons living in poverty, and because patient-provider communication has a clear and significant impact on patient outcomes such as adherence, satisfaction, and health status, interventions to increase patient-physician partnership are important strategies to overcome disparities in hypertension care and outcomes.

## Methods

### Study design and specific aims

#### Specific aim one

**Figure 1:**
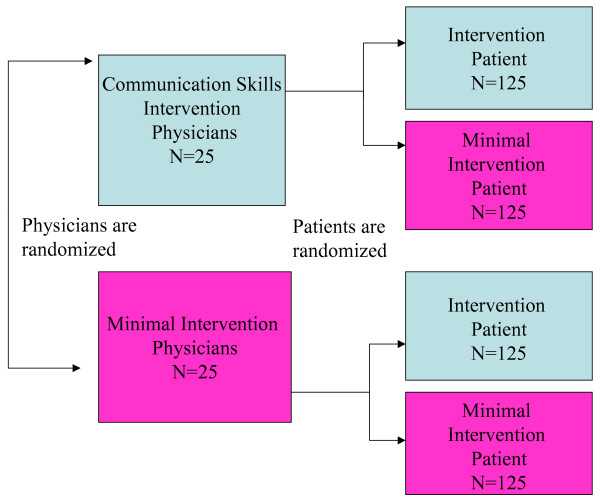
**Patient-Physician Partnership study design**. The study uses a 2 by 2 factorial design to simultaneously study the effect of physician communication skills training and/or patient activation by community health workers (CHWs). All physicians, including those in the minimal intervention, receive a copy of hypertension treatment guidelines and are videotaped with a simulated patient before randomization. The patient intervention includes coaching by CHWs and a photonovel. CHW contacts are 20 minutes at enrollment, 2 weeks, 3,6,9, and 12 months. All patients, including those in the minimal intervention, receive monthly newsletters.

Recruit 50 primary care physicians and 500 of their patients who have uncontrolled hypertension into the Patient-Physician Partnership (Triple P) study, a randomized controlled trial with a two-by-two factorial design, to simultaneously study the effect of a patient activation and/or a physician communication training intervention on adherence to recommended treatment for high blood pressure (Figure 1). Patients will include adults, aged 18 and older, who receive care in several urban community health clinics serving primarily African-American and low socioeconomic status populations.

#### Specific aim two

Compare the relative effectiveness of the patient and physician interventions, separately, and in combination with one another, with the effectiveness of minimal interventions by evaluating their impact on the following outcomes measured at enrollment, three months, and twelve months: 1) patient adherence to medication and lifestyle recommendations (appointment-keeping, prescription refill rates, and patient self-reports); 2) patient and physician ratings of quality of care (physicians' participatory decision-making (PDM) style and satisfaction); 3) patient-physician communication behaviors, including adherence-specific communication, measured pre- and post-intervention; 4) health outcomes, including blood pressure control; and 5) emergency room use and hospitalizations.

#### Specific aim three

Assess the moderating effects of patient and physician variables on the relationships between the intervention and the main outcomes. Important moderating patient variables include age, ethnicity, gender, health literacy, and physical and emotional health status. Moderating physician variables include age, ethnicity, gender, knowledge of hypertension management, clinical experience, psychosocial-mindedness, attitudes towards diversity, and previous training in communication skills.

We hypothesize that the combined patient and physician intervention will have the greatest effect on processes and outcomes, the patient and physician interventions separately will each have an intermediate effect, and the combined patient and physician usual care group will have no appreciable effect. Specifically, we hypothesize that compared to patients and physicians in the usual care group, patients and physicians in the intervention groups will have higher rates of patient adherence to therapeutic recommendations; higher ratings of partnership with physicians, quality of care, and satisfaction; more patient-centered communication behaviors by physicians as measured by audiotape; more communication across the participation continuum by patients as measured by audiotape; higher rates of blood pressure control; and lower rates of emergency room use and hospitalizations.

### Study population and settings

The Baltimore, Maryland metropolitan area has one of the five highest percentages of African Americans of the Standard Metropolitan Statistical Areas in the United States (U.S. Census Bureau. . Accessed on 24 July 2007). The Triple P study occurs in primary care sites affiliated with Baltimore Medical System (four sites), Johns Hopkins Community Physicians (five sites), Total Health Care (two sites), Jai Medical Group (three sites) and five other independent practice locations. These sites were chosen because they are community-based and serve a patient population that is primarily low income/and or ethnic minority (African-American). Approximately 60 to 100% of the patients in participating sites are African-American and 35 to 55% earn below 200% of the federally defined poverty guidelines.

### Recruitment strategies

#### Physicians

Letters co-signed by medical directors of each provider organization and the principal investigator (PI) introduced the study to physicians. The letter outlined the goals of the study, gave a general description of the interventions, and described the responsibilities of physicians caring for study patients. Physicians were also told that they would receive continuing medical education credits, tailored, individualized feedback regarding their interviewing skills, and $200 paid to them either individually or to their organization. The PI subsequently attended staff meetings to present the study to physicians and to answer any questions they had. After the presentation, physicians were given a sign-up sheet they could return immediately or by fax to the PI's office. Research staff made follow-up phone calls to physicians who did not respond by fax within two weeks of the presentation at each site. Practice leaders facilitated communication with the physicians at their sites.

#### Patients

Patients were recruited using two strategies. Initially, we obtained approval from the Johns Hopkins Institutional Review Board and the participating clinical sites to identify potentially eligible patients from claims data. All patients aged 18 and older with an ICD-9 diagnosis of hypertension (401.00 – 401.9), based on one or more claims in the past 12 months were eligible for consideration.

For each participating physician, if the physician's panel size of potentially eligible patients was 200 or less, we attempted to recruit all patients. If the panel size was greater than 200 patients and more than 50% white, we over-sampled ethnic minority patients by taking up to 140 minority patients and sampling white patients to add up to a total pool of 200 patients per physician. If the panel size was greater than 200 patients and less than 50% white, we randomly sampled 200 patients per physician.

We obtained patients' name, race, gender, and contact information, and compiled this information into an electronic database that was then used by research staff to mail letters that invited patients to participate in the study. The letter, sent on the letterhead of each participating clinical site, told patients that their primary care physicians had signed up for the project and that his/her patients with hypertension were being invited to participate. The letter also included a postcard that could be returned to study staff to indicate if the patient did not wish to be contacted further. If a refusal was not received from a given patient within two weeks, the letter was followed by a telephone call to tell them about the study, confirm eligibility and interest, and ask them if they would be willing to speak further about the study with a member of the study staff when they arrived at the clinic for their next appointment. If they agreed, they were asked to arrive one hour before their appointment, and the research assistant (RA) called them one to two days prior to their scheduled appointment to remind them.

Initially, we attempted to recruit at least 10 and no more than 15 patients per physician. Recruiters were told to call all of the patients in each physician's recruitment sample until this goal was achieved. We aimed to complete recruitment at certain sites prior to beginning recruitment at other sites in order to maximize staff efficiency. Towards the end of the recruitment phase, we adjusted our recruitment target to a minimum of five patients per physician.

After the enforcement of the Health Insurance Portability and Accountability Act (HIPAA), we obtained a Waiver of HIPAA privacy authorization from the Johns Hopkins IRB and entered into agreements with many of the health plans to allow data sharing for patient recruitment. However, some insurers were hesitant to share claims data with the team for the purposes of recruitment, as described above. Since this impacted our ability to recruit patients by telephone, we sought and obtained approval from the IRB to recruit patients onsite by sending research staff to participating sites on recruitment days agreed upon by research and clinical site staff. The recruitment process is the same for either strategy, except that patients identified by claims data and recruited by letter and telephone calls were prepared to arrive early for their appointment, while patients recruited onsite had less time before their appointment to complete the recruitment process. In the latter situation, research staff were instructed to complete data collection on only absolutely required items prior to the patient's appointment and to avoid any interference of routine clinical practice. We have recorded the recruitment strategy that was used for each patient and will examine its association with agreement to enter the study and with baseline demographic and clinical characteristics.

At the index visit, the RA assigned to data collection met the patient, described the study, obtained consent, administered the baseline interview, checked patients' seated blood pressures using standard techniques, and arranged for the patient's visit to be audiotaped. Patients were then randomized to a minimal intervention or a community health worker (CHW) intervention. Those randomized to the CHW intervention attended their first intervention visit for pre-visit coaching, and patients assigned to the minimal intervention group had a five-minute welcome to the study in which the RA provided an educational newsletter about hypertension.

#### Eligibility criteria

Physicians recruited for the Triple P study were general internists and family physicians who saw patients at least 20 hours per week at one of the participating study sites. Physicians were only excluded if they intended to leave the practice within 12 months of the beginning of the patient recruitment period. Patients recruited for the Triple P study were adults aged 18 years and older, had a diagnosis of hypertension (at least one claim with the ICD-9 code 401 in the preceding year), and were able to provide contact information for themselves and at least one other person and written consent to participate in the randomized clinical trial. Patients were excluded if they: 1) refused to give informed consent; 2) appeared to be too acutely ill, disoriented, or unresponsive to complete the baseline assessment (interview, blood pressure, weight measurement, and audiotaped visit), 3) stated that they had not been told by their doctor that they were hypertensive, 4) were likely to move away from the Baltimore area in the next 12 months; 5) were planning to change where they receive medical care within the next 12 months; 6) were currently involved in a disease management program, research program or study for hypertension, kidney disease, heart disease, or diabetes; or 7) if they had a medical condition that might limit their participation in the study over the next five years (*e.g*., AIDS/HIV, schizophrenia, cancer (except skin), Alzheimer's or other form of dementia; end-stage renal disease, congestive heart failure, or active tuberculosis). This information regarding medical conditions was obtained from claims data, when available, to exclude ineligible patients from the recruitment database, and ascertained by patient self-report during onsite recruitment.

#### Randomization

Randomization was conducted first at the physician level and then randomizing patients within physician groups. After obtaining informed consent and completing baseline data collection (background questionnaire and videotaped interview with the standardized patient), physicians were randomly assigned to the minimal intervention or communication skills intervention. The physician intervention was assigned stratifying by clinical site. Random blocks of size two and four were used, and a list of random numbers between zero and one was generated in Stata version 7.0 (Stata corporation, Texas, USA). Patients were randomly assigned to the minimal intervention or the community health worker intervention after confirming eligibility, obtaining informed consent, and completing the baseline patient interview. The patient intervention was assigned stratifying by physician using random blocks of size four. The study statistician generated the allocation sequence for both physicians and patients and placed the intervention assignment for each subject in opaque envelopes to be opened by research assistants after the subject had completed the baseline assessment. The sequence was concealed until after interventions were assigned.

Due to the nature of the interventions, complete masking of participants, investigators, and community health workers was not possible. However, all interviewers and community health workers (who collected data from patients) were masked to physician intervention assignment. Additionally, research interviewers at enrollment were masked to patient intervention assignment until after baseline data collection was complete, and research interviewers at follow-up interviews (different staff) were masked to patient intervention assignment until the end of the interview (when patients were asked to evaluate the intervention). Physicians were not informed of the intervention assignment of their patients.

### Interventions

#### Patient interventions

The intensive patient intervention was based on a model of patient education, characterized by pre-visit coaching, that has been shown to improve patients' communication with providers and health outcomes [36,37], and includes aspects of medical interviewing relevant to the participation continuum (engagement, activation, and empowerment). We chose community health workers to administer the intervention and developed a mechanism for ongoing reinforcement and support in order to enhance the cultural appropriateness, and thereby the sensitivity, credibility, relevance, and effectiveness of the intervention for minority patients.

The intervention integrates cognitive, behavioral, and affective programmatic elements in two stages. The first stage of the intervention is comprised of a 20-minute pre-visit coaching session by a CHW in a room at the clinical site immediately prior to the patient's index visit with his/her physician, followed by a 10-minute exit or debriefing session after the visit. The CHWs used a structured protocol in the pre-visit coaching session to accomplish the participation (engagement, activation, and empowerment) goals.

The second stage continues contact between the CHW and the patient through a series of 10 to 15 minute check-in telephone calls at two weeks, three months, six months, nine months, and twelve months from baseline. In addition, the CHWs were available to patients by phone on an 'as needed' basis over the one-year follow-up period. A specially crafted serial photonovel featuring an ongoing drama was mailed to patients to coincide with their telephone follow-up calls. In this photonovel, a CHW, patient, and primary care physician are portrayed dealing with the daily challenges of hypertension management within the broader context of the patient's life. Each issue of the photonovel conveys a specific theme common to hypertensive patients as they attempt to meet everyday challenges associated with the many aspects of hypertension self-management (*e.g*., stress, family and financial issues, medication side effects, diet, exercise, alcohol use, and adherence with appointments). The CHWs who were hired for this study lived in the communities served by some of the participating clinics, and they helped investigators to create the storyline, write the script, and take photographs for the photonovel. Use of photonovels in diverse populations has demonstrated their superiority to standard health education materials in terms of interest, credibility, and readability [38-40].

In addition, all patients (intensive and minimal intervention) receive a monthly newsletter that includes a question and answer column, a recipe exchange, health tips, and reminders to keep scheduled appointments. The newsletters are designed to meet the needs of low literate adult readers by not exceeding a fifth-grade reading level and through the presentation of information through a familiar engaging and friendly format.

#### Physician interventions

The intensive physician intervention is a continuing medical education (CME) communication skills training program based on models previously shown to be effective in improving physicians' interviewing skills and patient outcomes [41]. A critical component of this program is the individualized feedback that intervention physicians receive regarding their interview with a simulated patient. The program includes those aspects of medical interviewing relevant to the participation continuum in the areas of data-gathering, patient education and counseling, rapport-building, and facilitation and patient activation. Although ambitious in its scope, the CME is designed for convenience and ease of administration. The estimated time for administration is approximately two hours, during which the physician reviews his or her personal interview with the simulated patient and completes workbook exercises.

Briefly, the CME is comprised of an interactive CD-ROM that is created using a videotape of each study physician's interview with a simulated patient collected at baseline. This patient is an African-American man with hypertension scripted to present common barriers and culturally specific beliefs and expectations related to adherence with hypertension therapy. The videotape is saved to the CD-ROM within a software program that shows the categorization of every statement spoken by either the patient or the physician. The coding scheme, called the Roter Interaction Analysis System (RIAS), is a widely used approach to the assessment of medical visit communication [41-44]. The software allows the physician to navigate the interview in an efficient manner and quickly review examples of specific skills. Specifically, physicians may go directly to those parts of the visit that interest them; see a visual summary of their conversation with the patient over the course of the visit; review the different kinds of talk that comprise the conversation and select samples of the talk, by category, for review; and view and listen to video-glossary examples of talk categories and proficiencies that are useful in improving patient adherence to hypertension treatment.

A workbook that accompanies the CD-ROM directs physicians to the primary features of the software; provides an orientation to the RIAS analysis approach; and includes case-based exercises to be completed by the physician. These exercises include a review of their skills in five areas for improving patient adherence (eliciting the full spectrum of patient concerns; probing patients regarding their knowledge and beliefs about hypertension; monitoring patient adherence; assessing obstacles and resources, and eliciting a commitment to the therapeutic plan). The workbook and CD-ROM also review the four functions of the medical interview (data-gathering, patient education and counseling, rapport-building, and facilitation and patient activation) with the corresponding communication skills. Completion of the workbook and an evaluation form provides documentation of the physician's completion of the CME program.

All physicians (intensive and minimal intervention groups) receive a copy of the JNC-VII hypertension treatment guidelines at baseline and a monthly newsletter with study updates and summaries of recent journal articles related to cardiovascular care and/or health disparities.

### Data collection

In addition to the main outcome and process measures, we collected data at baseline to define the characteristics of the study subjects and to describe the characteristics of experimental groups after randomization. While the intervention status is the main predictor variable, we measured other factors known to be predictors of adherence and blood pressure control (potential confounders and effect modifiers) and factors that could explain why the intervention did or did not work. We selected instruments that are generally relatively brief, have been used successfully in primary care settings, and have been shown to be reliable and valid in inner city ethnic minorities and persons living in poverty. Table 1 shows the sociodemographic and attitudinal variables, self-reported adherence, health service utilization, healthcare process, and health outcome measures collected from patients at baseline and over follow-up. Table 2 shows the variables collected from physicians at baseline, post-intervention, and the end of the study.

**Table 1:** Schedule of Variables Collected from Patients in Patient-Physician Partnership Study

**Measurement/Collection Method**	**Index visit**	**3 months**	**12 months**
**Questionnaires**			

Sociodemographics (age, sex, race/ethnicity, education, income, occupation, health insurance)	X		

Attitudes, beliefs, and behaviors (trust/mistrust, health behaviors, problem solving*, self-efficacy, spirituality, self-reported adherence to medications and lifestyle recommendations (HBS), perceived susceptibility to illness*, health literacy**)	X	X	X

Health Status (physical and mental, measured by MOS-SF12 & CES-D), Healthcare utilization* (emergency room visits and hospitalizations), Healthcare process (perceptions of biased care, trust, respect, PDM with physicians, visit-specific and overall satisfaction)	X	X	X

**Physical Examination **(BP, BMI)	X	X	X

**Blood laboratory measures **(Cr, eGFR, HbA1c, Hb, CaPO4, lipids)		X	X

**Urine laboratory measures **(microalbuminuria)		X	X

**Audiotapes **(patient-physician communication)¶	X		

HBS = Hill-Bone Adherence Scale, CES-D = Center for Epidemiologic Studies Depression Scale, PDM = participatory decision making; BP = blood pressure; BMI = body mass index; Cr = creatinine; eGFR = estimated GFR using MDRD equation; Hb = hemoglobin;

* = not measured at baseline; ** = measured only at baseline; ¶collected after first patient intervention

contact and after physician intervention.

**Table 2:** Schedule of Data Collected from Physicians in the Patient-Physician Partnership Study

	Baseline	End of study
Demographics (age, gender, race, ethnicity, place of birth, residency training, board certification status, practice experience)	X	

Specialty (Internal Medicine or Family Medicine)	X	

Previous Communication Skills CME Training	X	X

Previous Hypertension CME Training	X	X

Attitudes about Race*	X	X

Self-reported communication and PDM style	X	

Job stress and satisfaction	X	

Self-efficacy in managing adherence problems, hypertension, and patients from socially and culturally diverse backgrounds	X	X

	Pre Intervention	Post Intervention

Videotape with simulated patient	X	

Audiotapes with 5–10 hypertension patients		X

Visit-Specific Satisfaction with each patient		X

Perceptions of patients' social and behavioral characteristics		X

Use/process evaluation of CD-ROM/Workbook **		X

*Explicit attitudes measured at baseline before index visit; implicit attitudes measured only at end of study using the Implicit Association Test (IAT); ** Intervention physicians only

### Main outcome measures and statistical analysis plan

Randomly assigned treatment group (physician and patient intervention, physician intervention only, patient intervention only, or physician and patient minimal intervention) is the main independent variable for this study. All efficacy analyses will be performed using the 'intention-to-treat' principle. Clinic site was a stratification variable for randomization and is expected to be balanced across treatment groups by design. Descriptive statistics are used to summarize patient and physician characteristics at baseline. Comparability of patient groups after randomization will be assessed with regard to pre-intervention sociodemographics, health status measures, use of medical services in the previous six months, patient preferences for involvement in care, and other key variables. The comparability of physician groups after randomization will be determined based on physician sociodemographic data as well as pre-intervention measures of training and self-efficacy regarding management of hypertension, non-adherence, and social and culturally diverse patients.

The main study outcomes are measures of adherence to recommended treatment. Because there is no gold standard for what defines satisfactory versus poor adherence, measurement of adherence is multifaceted and includes appointment-keeping, pharmacy records, and subjective perceptions (patients', providers', and CHWs' report of patient compliance). These indicators tap different dimensions of the compliance challenge and reflect varied levels of patient effort and commitment and measurement rigor. Our primary outcome, upon which our sample size is calculated, is appointment-keeping. The number of primary care appointments scheduled and kept will be tracked using clinic schedules and claims data. Broken appointments that have been rescheduled and kept within a two-week window are considered a kept appointment; broken appointments that are rescheduled but not kept, or rescheduled outside of the two-week window are considered a missed appointment. Patients are also asked about the number of ambulatory visits (primary care and medical subspecialty) occurring within the previous six months at their three- and twelve-month follow-up interviews, and we will compare these self-reports to the information obtained from administrative data.

Change in systolic and diastolic blood pressure and blood pressure control status will be examined as secondary outcomes. Blood pressure (BP) is measured by trained and certified observers using an automatic oscillometric monitor (Omron HEM 907). This device programs a five-minute delay before activation and has a 30-second delay between the triplicate measurements. We will use two measures – the average of all three measurements and the average of the last two measurements – obtained at each time point (before randomization, at three months, and at twelve months of follow-up). BP control is dichotomized as uncontrolled (SBP ≥ 140 mmHg or DB P ≥ 90 mmHg) or controlled (SBP <140 mmHg and DBP <90 mmHg).

The data on outcome variables fall into two broad categories: dichotomous data, such as blood pressure control, yes or no; and continuous variables, such as the percentage of appointments scheduled and kept within a two-week period), compliance score (from Hill-Bone Compliance to High Blood Pressure Therapy Scale) [45], patient-centered interviewing score (obtained from audiotape analysis of patient-physician communication behaviors using the RIAS) [42-44], or participatory decision-making score [22]. In addition to the nature of the study variables, two additional design factors need to be considered in the data analysis stage. First, the trial naturally originates repeated measurements over the one-year follow-up [46-48]. Although we will compare study endpoints at the three- and twelve-month follow-up visit, this analysis is inefficient because it does not simultaneously use all available information, and at the same time it is subject to the multiple comparison problem [46]. We will use two approaches to analyze repeated measurement outcomes. First, for variables such as appointments scheduled and kept within a two-week period, we will compute a summary measure across time for each subject (in this case, the percentage of appointments scheduled and kept) [46,49]. This approach is, under a wide variety of circumstances, almost as efficient as other analyses of repeated measurements, and it provides a simple descriptive outcome for each participant that incorporates all time-dependent information. From a conceptual perspective, such summary measures are justified because all appointments are equally important throughout follow-up. In addition, it is relatively straightforward to incorporate data from losses to follow-up into summary measures for the main outcome variable: for each subject lost to follow-up, we can estimate that he should have had at least two visits over a four-week interval to bring his blood pressure under control, and then he should have had at least one visit every three months for the rest of the study period. This allows us to obtain a summary measure of adherence from each study participant, even if he/she is lost to follow-up shortly after randomization. Second, in addition to obtaining summary measures over time of key dependent variables, we will also use generalized estimating equations (GEE) to model the marginal expectations of the outcome variables as a function of randomized assignment [46,50,51]. The GEE approach takes into account the correlations of the data derived from the same participant, but the model coefficients are consistent even if the covariance structure of the outcome variable is incorrectly specified.

The second methodological consideration in this study is derived from the fact that one of the randomized factors applies to participating physician, rather than to patients. In practice, then, study patients are nested within physicians. Because of this 'multilevel' structure and the potential importance of group-level attributes in influencing individual-level outcomes, we will analyze the data using hierarchical models, also known as multilevel, random-coefficient, or covariance component models [52-54]. These models can be conceptualized as two-stage systems of equations, in which the individual variation within each group is explained by an individual-level equation, and the variation across groups in the group-specific regression coefficients is explained by a group-level equation. The main independent individual-level variable will be the randomized patient assignment, and the main independent group-level variable will be the randomized physician assignment. The hierarchical models allow for the simultaneous consideration of patient-specific and physician-specific explanatory variables as well as for the study of interactions between variables at patient and physician levels.

To guide our estimations of sample size and power, we used the data from a previous meta-analysis of the evaluation of effectiveness interventions to improve patient adherence to estimate clinically relevant and feasible treatment effects for the interventions and outcomes studied [55]. In this meta-analysis, the overall effect size for interventions for appointment keeping, measured as a function of the standard deviation of the outcome measure (Cohen's *d*), ranged from 0.40 to 0.70. We estimated our sample size to detect as significant an effect size (in standard deviation units) of 0.40, with 90% power and a probability of type I error of 0.05 (two-sided). We also assumed that the nesting of patients within physicians would introduce some within physician correlation that would decrease the efficiency of our estimators by about 30%. Under these assumptions, the estimated sample size needed was 240 patients per group, for a total of 480 patients and 48 participating physicians. We planned to enroll 50 physicians and 500 patients.

### Ethics and Consent

The trial received approval from the Johns Hopkins Institutional Review Board. Informed written consent was obtained from all participating physicians and patients. Subjects were free to withdraw from the study at any time, to refuse to answer any question, and to either stop audiotaping or to have audiotapes of any visit dropped from the study. Confidentiality of the study data was maintained as follows: none of the patient information was released to their physician, health care organization, or any other party without the patients' permission. Phone contacts to locate the study subject did not suggest the content of the study. All study data were stored in locked file cabinets at Johns Hopkins and not the clinical sites. Personal identifiers were removed as soon as possible. Audiotape data were transferred onto CD-ROMs for coding purposes, and stored in locked files after identifiers were removed. A code key is kept in a separate location restricted to the principal investigator and project director. Each organization received an incentive of $200 per participating physician and each patient received $25 for completing each interview/exam (a maximum of $75 for completing the baseline, three-month, and twelve-month assessments).

### Baseline characteristics of study sample

#### Baseline characteristics of the physicians

**Figure 2:**
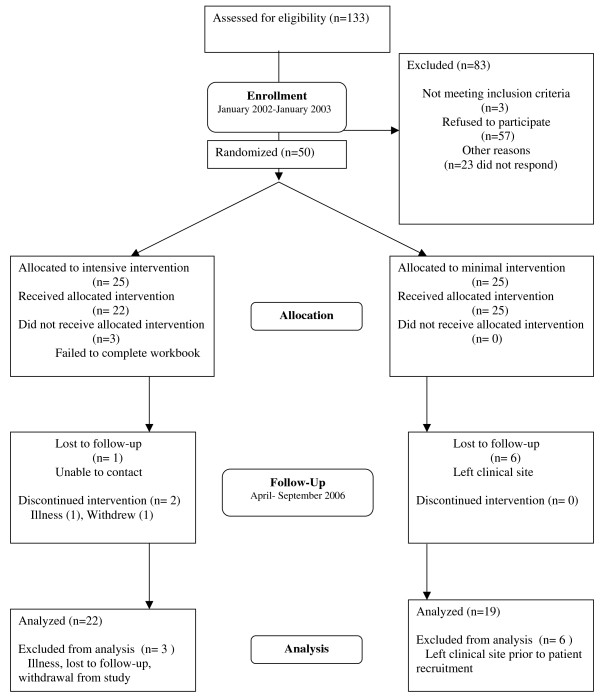
**Patient-Physician Partnership study CONSORT flowchart for physicians**.

Physicians were enrolled between January 2002 and January 2003. We contacted 133 physicians, of whom 110 responded; 23 physicians did not respond despite several phone calls, faxes, and emails from the study. Fifty-three physicians agreed and 57 refused, citing lack of time or interest. Two of the 53 physicians who agreed to participate left their clinical site before baseline data collection, and one physician was determined to be ineligible because she had no primary care patients and delivered only urgent care.(Figure 2) Characteristics of the 50 physicians recruited to the study are shown in Table 3. They were mostly general internists (74%) with a mean age of 43.0 years and mean practice experience of 11.9 years. Just over half (52%) were women, and they were ethnically diverse. Most were very confident in their ability to care for socially disadvantaged (60%), ethnic minority (70%), and hypertensive patients (82%); however, only a third (34%) were confident in their ability to care for non-adherent patients.

**Table 3:** Patient-Physician Partnership Study: Demographic and Baseline characteristics for n = 50 physicians

Characteristic	No. of Physicians (%)	Mean (standard deviation)
Age, years		43.0 (9.3)
Women	26 (52)	
Ethnicity		
African American	16 (32)	
Asian	10 (20)	
White	19 (38)	
Hispanic/other	2 (4)	
Practice experience, years		11.9 (8.4)
Internal medicine	37 (74)	
U.S. medical graduate	37 (74)	
Board certified	45 (90)	
CME in communication skills	21 (42)	
CME in hypertension	31 (63)	
Very confident caring for:		
Socially disadvantaged	30 (60)	
Minority patients	35 (70)	
Hypertensive patients	41 (82)	
Non-adherent patients	17 (34)	
Strongly agree:		
Communicate effectively	15 (30)	
Gain patients' trust	7 (14)	
Patients as partners in treatment	8 (16)	

#### Baseline characteristics of patients

**Figure 3:**
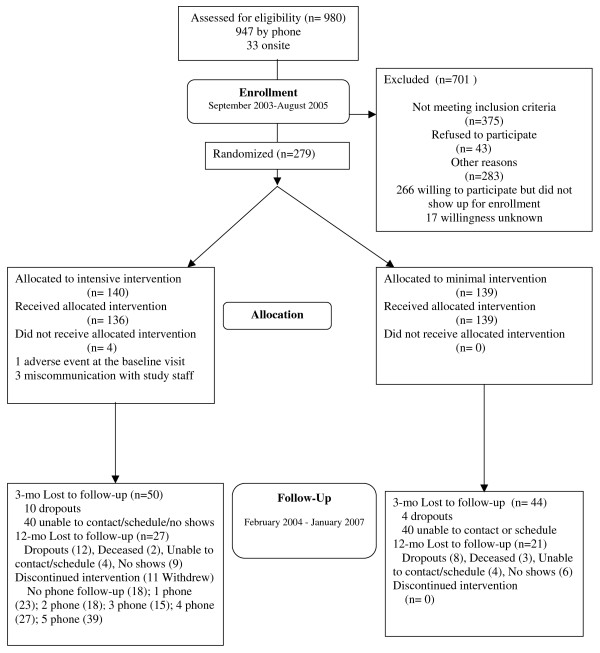
**Patient-Physician Partnership study CONSORT flowchart for patients**.

Patients were enrolled between September 2003 and August 2005. We sent letters to 9,077 patients who were identified by claims data to be potentially eligible. Of these patients, 287 letters were returned undelivered, and 908 patients refused by a mail-in post card. Research staff attempted calls to or approached onsite 3,240 patients. No attempt was made to call the remaining 4,642 of these patients for several reasons, including: 1) their physician withdrew from the study before patient recruitment was complete; 2) the targeted number of patients for the targeted physician was already scheduled; and 3) the health plan to which the patient belonged would not allow research staff to call until the patient had signed a HIPAA privacy authorization form and returned it to the office manager first. Of the 3,240 patients for whom calls or contact was attempted, 1,375 patients were contacted (by either by phone or in-person onsite), and 1,865 patients were not contacted (*e.g*., patient was deceased, phone disconnected, no answer, left message, busy, or wrong number). There were 395 patients for whom eligibility was not assessed because the patient refused immediately when approached. Eligibility was assessed for 980 patients. Figure 3 shows the recruitment outcome for the 980 patients for whom eligibility was assessed. Table 4 shows baseline characteristics of the 279 patients who enrolled in the study. These patients were 61.3 years on average; 66% were women and 62% were African American. The average number of years of education was 11.8 years, but only 19% were employed full-time, and 70% of the sample reported an annual household income of less than $35,000. Ninety percent had health insurance and 92% had prescription drug coverage. Diabetes was the most common co-morbid medical condition (44%), followed by depression (24%), and coronary heart disease (17%). The sample had a mean body mass index of 32.9, and 48% had controlled blood pressure using JNC-7 criteria. Table 5 shows patient reports of self-reported adherence, physicians' participatory decision-making, and satisfaction with care at baseline.

**Table 4:** The Patient-Physician Partnership Study: Baseline Demographic and Clinical Characteristics for 279 Patients

Characteristic	No. of patients (%)	Mean (standard deviation)
Age, years		61.3 (11.8)
Gender, female	184 (66.0)	
Race		
African American	173 (62.0)	
Asian	3 (1.1)	
White	101 (36.2)	
Marital status, married	98 (35.4)	
Education		
< High school graduate	87 (31.3)	
Years		11.8 (2.4)
REALM, ≥ 9^th ^grade	173 (62.9)	
Income		
< $10,000	98 (37.7)	
< $35,000	170 (70.0)	
Employed		
Full time	51 (18.6)	
Part time	16 (5.8)	
Retired	96 (35.0)	
Disabled	59 (21.5)	
Healthcare insurance	249 (90.0)	
Medicaid	85 (30.7)	
Medicare	107 (38.9)	
Other	140 (50.9)	
Prescription plan	257 (92.8)	
MOS-SF-12 physical component		40.3 (12.2)
MOS-SF-12 mental component		50.5 (10.9)
Comorbid medical cond.		
Diabetes	121 (44.0)	
CVD	48 (17.4)	
Angina	25 (9.2)	
Heart failure	16 (5.9)	
Stroke	15 (5.4)	
Kidney failure	10 (3.7)	
Depression	64 (23.5)	
Body mass index		32.9 (8.1)
Systolic blood pressure		135.3 (19.4)
Diastolic blood pressure		75.9 (12.9)
Blood pressure control (JNC-7)	130 (48.0)	

**Table 5:** Patient-Physician Partnership Study: Baseline Adherence, Participatory Decision Making and Satisfaction for 279 Patients

Characteristic	No. of Patients (%)	Mean (standard deviation)
Hill-Bone Scale		
Sodium subscale		5.4 (1.6)
Appointment subscale		2.7 (1.0)
Medication subscale		10.3 (2.0)
Total		18.4 (3.0)
		
Medication non-adherence*	98 (36.4)	
		
Participatory Decision Making		69.7 (23.3)
		
Satisfaction:		
Satisfied with visit		
Neutral to strongly disagree	4 (1.5)	
Agree	139 (50.9)	
Strongly agree	130 (47.6)	
Would recommend MD		
Neutral or disagree	3 (1.1)	
Agree	187 (68.5)	
Strongly agree	83 (30.4)	

*Medication non-adherence (Morisky) = a positive response to at least one of four questions regarding forgetting to take medications, stopping medications because of feeling better, stopping medications because of feeling worse, and missing medications because of carelessness.

** Participatory Decision-Making is measured using patient ratings of physicians' likelihood of giving the patient choice, control, responsibility in decision-making and scored on a 0–100 point scale.

## Discussion

This study has several strengths and as such, its expected impact is significant. There is strong evidence that patient-centered communication behaviors impact upon patient adherence, patient satisfaction, and important health outcomes. Because racial, ethnic, and social class disparities in health care exist across disease conditions and types of care (preventive, diagnostic, and therapeutic procedures), this suggests that fundamental aspects of healthcare, such as patient-provider communication, may play a role. Furthermore, studies show that ethnic minority patients experience lower levels of patient-centered communication and greater verbal passivity with physicians than whites and patients with higher levels of education [56,57].

Communication skills programs that prepare health care providers to deliver high quality interpersonal and technical health care to an ethnically and socially diverse population are a promising mechanism by which disparities in health care may be reduced. Additionally, culturally targeted patient interventions that increase engagement, activation, and empowerment among ethnic minorities and persons living in poverty are likely to increase patients' ability to: 1) fully participate in the medical interview, 2) negotiate treatment plans by engaging in joint problem-solving and collaborative treatment decision-making with physicians, 3) adhere to treatment and management recommendations, and 4) improve health outcomes. We have incorporated several successful features of previous interventions in ethnic minority and socio-economically disadvantaged populations in our proposed study as well as a number of novel elements. We propose the use of multifaceted (educational, behavioral, and affective) intervention approaches, incorporating culturally and linguistically appropriate methods tailored to individuals' needs (*e.g*., the use of community health workers as interventionists to address common cultural beliefs and practices, the development of a participatory photonovel that is engaging and user-friendly) to support the therapeutic partnership from both the patient and physician perspective. It is also expected that involvement of practice leaders in all aspects of study design, intervention development and implementation, and participant recruitment and follow up, will enhance the external validity of this study.

Limitations of the study include: 1) loss to follow-up among randomized physicians, which affected the number of patients that could be enrolled into the study; 2) failure to reach the recruitment target among patients, which may reduce the study's statistical power to detect differences in the primary outcome, 3) the lack of repeated exposures to the intervention for physicians and the reliance on telephone follow-up for all contacts except the first intervention contact for patients; 4) the relatively small percentage of the sample that had uncontrolled blood pressure at baseline (52%); and 5) variability in the accessibility and quality of administrative data from the large number of health plans.

Nonetheless, because it addresses many limitations of previous studies, The Patient-Physician Partnership to Improve High Blood Pressure Adherence will provide new knowledge about how to improve patient adherence, quality of care, and cardiovascular outcomes and how to reduce disparities in care and outcomes of ethnic minority and poor persons with hypertension.

## Competing interests

The authors declare that they have no competing interests.

## Authors' contributions

LC, DR, LB, EM, MB, and DL conceived of and designed the study. LC, DR, SL, EM and KC participated in the analysis and interpretation of data. KC provided statistical expertise. LC and DR drafted the article. All authors read and approved the final manuscript.
